# The relation between leadership styles in higher education institutions and academic staff’s job satisfaction: A meta-analysis study

**DOI:** 10.3389/fpsyg.2022.1038824

**Published:** 2022-11-17

**Authors:** Gamze Kasalak, Beysun Güneri, Vesile Ruya Ehtiyar, Çiğdem Apaydin, Gulay Özaltın Türker

**Affiliations:** ^1^Department of Educational Sciences, Akdeniz University, Antalya, Turkiye; ^2^Department of Social Sciences, Muğla University, Muğla, Turkiye

**Keywords:** leadership styles, job satisfaction, academic staff’s job satisfaction, meta-analysis, higher education institutions

## Abstract

In this study, it is aimed to examine the relationship between leadership in higher education institutions [HEIs] and academic staff’s job satisfaction, which is formed by combining different leadership styles in higher education institutions, using the meta-analysis method based on correlational research. For this purpose, it was investigated whether there was a significant difference between the effect sizes of the studies investigating the relationship between leadership in HEIs and academic staff’s job satisfaction between the years 2010–2022, according to the moderator variables (leadership styles, continent, culture, and Human Development Index [HDI]). A total of 57 research data, including sample size and Pearson correlation coefficient data, were evaluated within the scope of the research. Correlational studies were calculated according to the random effect model in terms of effect direction and overall effect size; The estimated effect size value was found to be 0.374. This value shows that the overall effect size of the relationship between leadership in HEIs and academic staff’s job satisfaction is positive and moderate. However, there is no significant difference between the effect sizes of the research examining the relationship between leadership styles in HEIs and academic staff’s job satisfaction, according to continent, culture and HDI moderator variables.

## Introduction

### Problem statement

New approaches to leadership in higher education are explored as universities face the challenges of competing in a globally competitive world while designing opportunities to build and develop sustainable leadership. While similar challenges exist in all industries, higher education is uniquely positioned given its role in developing new knowledge and disseminating existing knowledge (Jones et al., 2012). Universities provide qualified human capital by leading research activities to draw the attention of many institutions to the unresolved problems or weak areas of society, and also help the development of almost every sector effecting the economy. Therefore, universities need educational leaders who can fulfil their duties with the highest efficiency, integrity and the highest ethical standards in order to achieve their goals. Education leaders have many responsibilities including research, supervisory, administrative roles, job placement, supervision, event management and oversight of extra-curricular activities (Akhtar et al., 2021). However, Javed et al. (2020) state that responsibility is largely dependent on the leader and is subjective. According to the authors, to whom and what the leader is responsible for is subjective matter.

The changing demand for higher education challenges traditional assumptions not only about the nature, purpose, and place of higher education in society, but also about the most appropriate management and leadership systems that should operate in educational institutions. For example, Bolden et al. (2012) compares the traditional university model as a community of academics with a highly democratic and decentralized decision-making process that represents leadership as a shared responsibility with the increasingly common institutional or entrepreneurial approaches to leadership and management in universities. In recent studies, it has been examined how various leadership styles in higher education affect quality effectiveness, commitment, perception of organizational support, citizenship, and satisfaction in organizations (Alonderiene and Majauskaite, 2016; Sharma et al., 2016; Syakur et al., 2020; Öztürk and Kılıçoğlu, 2021).

To investigate current trends in higher education research, Tight (2012) analysed various higher education articles published between 2000 and 2010, he found an increase not only in quantity but also in quality of publications. Gumus et al. (2018) examined the leadership trends in educational organizations between the years 1980–2014 in their bibliometric study, and they found that the overall rate of the study group at the level of about 10 percent. It is observed that in the humanistic leadership theories period, leadership studies in higher education institutions are subjected to various leadership areas such as collaborative and distributed leadership (Youngs, 2017); transactional leadership (Sims et al., 2021); responsible leadership (Akhtar et al., 2020), instructional leadership (Shaked, 2021); transformational leadership (Sathiyaseelan, 2021); ethical leadership (Gok et al., 2017) and servant leadership (Dahleez and Aboramadan, 2022). The issue of leadership in higher education institutions, especially whether different leadership styles exist in higher education institutions, whether they are necessary, and whether the same theory and application framework is valid for the higher education sector as in other institutions (Siddique et al., 2011; Amzat and Idris, 2012) brought it to the fore. Because, as a large institution, a university is managed by various structures and administrative bodies, from the Rector, Vice-Rectors and Deans to academic councils, department managers, and administrative boards. Therefore, leadership styles in higher education institutions refer to different management roles and titles, from strategic management to managerial roles, transformational and visionary roles (Settles et al., 2019). It can be concluded that the roles of leaders in higher education can be complex and varied. Li et al. (2022) support this by emphasizing the complexity of the roles of education leaders in higher education, stating that they are responsible for fulfilling a variety of tasks from educational visionary to legal oversight. Apart from this, job satisfaction of lecturers is another important variable in order to increase the quality of education and training and to create university performance at universities. As suggested in limited research, appropriate leadership styles in higher education can increase the job satisfaction of academic staff (Alonderiene and Majauskaite, 2016). When leaders in HEI exhibit leadership characteristics and actions consistent by encouraging the job satisfaction, they positively affect many factors such as employee retention (Harris et al., 2016), organizational justice and organizational trust (Dahleez and Aboramadan, 2022), organizational commitment (Mwesigwa et al., 2020), academic staff performance (Jameel and Ahmad, 2020). Nguyen et al. (2021), found a high correlation between leadership and job satisfaction, and state that the leader style is important. Shaari et al. (2022) found a relationship between transformational and transactional leadership and job satisfaction in their research on academic staff. Therefore, this research focuses on the effect of leadership styles in HEIs on academic staff’s job satisfaction.

There are various meta-analysis studies investigating the effects of leadership style on job satisfaction in educational organizations in resent studies (Cakmak et al., 2015; Coğaltay et al., 2016). However, as a result of the literature review, no meta-analysis study was found that examines the effect of leadership style in higher education institutions on the job satisfaction of academic staff. It can also be stated that leadership studies in HEIs are less studied compared to leadership styles in primary, secondary and high school education institutions. It can be stated that the importance given to leadership in HEIs has increased significantly in recent years (Belias and Koustelios, 2014). Therefore, this research, focuses on the effect of leadership in HEIs on job satisfaction of academic staff, is expected to contribute to the literature. In addition, it is thought that the research will provide an opportunity to explain how leadership in HEIs affects the job satisfaction of academic staff.

This study makes an important contribution to the literature, as it is the first research to examine leadership styles in HEIs and academic staff’s job satisfaction through meta-analysis method. Although there are many empirical studies in the literature (Okan and Akyüz, 2015; Kiplangat et al., 2017), there is no study that clearly reveals the direction and effect of the relationship between leadership styles in HEIs and academic staff’s job satisfaction using the meta-analysis method. Although various empirical studies have been conducted to date, this study is summarized for the first time by combining the studies done so far with the psychometric meta-analysis method. In this context, the results of the relations obtained in the literature have been clearly revealed and a contributed the literature. Because, by bringing together the studies that deal with the relationships between these variables, it will be possible to determine the direction and strength of the relationships, and it will be possible to contribute to the literature. In addition, it will contribute to the clearer understanding of the relationship between leadership styles in HEIs and academic staff’s job satisfaction by researchers in the field. In summary, the study, and its results both contribute to the knowledge of literature and draw attention to the importance of increasing leadership styles studies in higher education institutions.

## Literature review

Universities have its own challenges (Anthony and Antony, 2017) because of having complex structure and uncertain decision-making processes (Hendrickson et al., 2013) which reveals the need for different leadership styles (Gigliotti and Ruben, 2017). In this context, it can be mentioned that the concept of leadership styles exists because there is a need for leadership in the management of higher education institutions.

According to Anthony and Antony (2017), leaders in HEIs encourage academic staff towards their academic work and can create social networks among academic staff. In addition, leaders in HEIs follow the mission of the university with a visionary approach; as entrepreneurs, risk-taking and flexible individuals, they can create structures to support change and affect the culture and values of HEIs (Anthony and Antony, 2017). It is also stated that leaders in higher education institutions are charismatic individuals who can foresee difficulties or opportunities, adapt to change, and do not hesitate to work to become stronger individually and professionally (Asaari et al., 2016; Thompson and Franz, 2016). In addition, as a reflection of leadership in HEIs, strategy, ethics, professionalism, goal orientation, experience, passion, recognition, and self-confidence are also emerging (Iordache-Platis, 2016). Since leaders in HEIs is associated with positions such as rector, dean, director, and head of department, academic leaders organize training programs, make planning in academic units, recruit academic staff, and evaluate and coordinate the institution (Hacifazlioglu, 2010). Mamiseishvili et al. (2016), on the other hand, state that especially department heads encourage productive behaviours through strong leadership roles in HEIs and are seen as a source that provides development opportunities as a model for other academic staff. Leaders in HEIs play a fundamental role in ensuring effective communication and thus building trust and transparency (Gigliotti and Ruben, 2017). In summary, leaders in HEIs are used in this research to refer to individuals who work as permanent academic staff in higher education institutions and who assume leadership and management roles within the university system (Morris and Laipple, 2015; Iordache-Platis, 2016) and it is related to the tasks or behaviours performed by the academic staff in the managerial position (Pani, 2017).

Leaders in HEIs directly or indirectly influence the academic world by using their unique experiences, teaching, and research skills (Thompson and Franz, 2016). One of the important variables affecting the academic world is job satisfaction. Job satisfaction is defined as the emotional reactions of employees towards their jobs and how they feel towards their jobs and organizations (Spector, 1997) and is associated with increasing employee behavior, motivation, and productivity (Bhuian and Islam, 1996). Leaders, with their knowledge and abilities, have an impact on the job satisfaction of the employees due to their features such as gathering people around certain goals and activating them to realize these goals (Eren, 2001) and being able to transfer their feelings and thoughts to the employees strongly (Goleman, 2002).

### Research hypothesis

Leaders are the role models of their subordinates within an organization. Various negative behaviours exhibited by leaders (for example, hiding information from subordinates; presenteeism) may also negatively affect their behaviour (Dietz et al., 2020; Akhtar et al., 2021). Therefore, it is extremely important for leaders who are role models to exhibit positive behaviour. Thus, employees will create an environment of creativity where they can improve their services, generate new ideas and encourage new ways of working (Karatepe et al., 2020). Similarly, given that academic staff take their leaders as role models, academics can pay attention to whether their own values are in line with the values displayed by the leaders in their institutions (Lee et al., 2017). It is expected that the job satisfaction of academicians who exhibit leadership styles appropriate to their own values will be positive. In a limited number of studies, it is stated that there are positive and significant relationships between leadership styles in higher education and job satisfaction of academic staff (Schulze, 2006; Lan et al., 2019). Based on this, the following hypotheses were developed in the research:

*H1*: There is a positive relationship between leadership in HEIs and academic staff’s job satisfaction.

The relevant literature shows that different styles of leadership in HEIs have an impact on the job satisfaction of academic staff, either directly or through intermediary factors (Alonderiene and Majauskaite, 2016; Dalati et al., 2017; Barnett, 2018; Rahman, 2018; Suong et al., 2019; Mwesigwa et al., 2020; Djaelani et al., 2021).

One of the important leadership styles that affect the job satisfaction of academic staff from research variables is transformational leadership. Transformational leadership is a process that changes the values, beliefs, and attitudes of its followers (Riggio, 2014) and aims to increase the self-confidence of individuals by revealing their talents and skills (Eren, 2015). In this context, transformational leadership draws a framework for the transformation of knowledge in HEI (Basham, 2012; Cetin and Kinik, 2015). A transformational higher education leader can increase job satisfaction by gaining the respect of the academic staff, considering the moral and ethical consequences of decisions, and giving individual incentives to increase the motivation of academic staff (Bass et al., 2003). Therefore, it is thought that transformational leadership in higher education may have a positive effect on academic staff’s job satisfaction. According to the research conducted by Mwesigwa et al. (2020) shows that transformational leadership styles positively affect the job satisfaction of academic staff. It is also stated in the same study that job satisfaction tends to increase when they provide better and more suitable working conditions by giving academic staff the freedom to take decisions, provide them opportunities to develop themselves with additional training programs, support their career development by counselling, reward them with incentive programs, provide fringe benefits, empower them and encourage their participation in some studies and some projects (Mwesigwa et al., 2020). In related studies, it has seen that there are positive and significant relationships between job satisfaction and transformational leadership styles (Robyn and Preez, 2013; Ali et al., 2014; Suong et al., 2019; Jameel and Ahmad, 2020).

Another style of leadership that positively affects academic job satisfaction is transactional leadership (Suong et al., 2019; Jameel and Ahmad, 2020). In transactional leadership, where the authority of the leader is dominant, the successful completion of tasks and follower harmony are emphasized through contingent rewards (Northouse, 2018). In this context, it can be mentioned that transactional leadership in HEIs uses reward or punishment to direct and maintain the extrinsic motivation of academic staff (Zheng et al., 2019). As a result, a transactional leader who clearly expresses the expectations in higher education institutions and promises awards and status to the academic staff if these expectations are met can positively affect the job satisfaction of the academic staff (Bateh and Heyliger, 2014).

In passive leadership, it is said that the leader avoids taking responsibility, refrains from making decisions, does not give feedback, and makes little effort to help his followers to meet their needs (Northouse, 2018). In passive leadership, it can be emphasized that leaders in higher education institutions are passive, ineffective, and unwilling or incapable of making decisions on their own when they lack knowledge, experience, and expertise. As a result, this leadership may negatively affect the job satisfaction of academic staff, as it causes lack of motivation and role ambiguity in academic staff (Belias and Koustelios, 2014).

It is important for academic staff to be aware of the existence of a servant leader who consider their views into account, loves, and respects them, understands, supports and exalts them (Yukl, 2018). However, increasing love, trust, and appreciation among teaching staff can be supported by spiritual leadership. In this way, spiritual leaders are a source of inspiration for the high performance of the academic staff, increase cooperation and encourage learning together (Yukl, 2018). Therefore, according to relevant literature examining the relationship between servant leadership (Alonderiene and Majauskaite, 2016), spiritual leadership (Wong et al., 2015; Djaelani et al., 2021) and job satisfaction, it can be concluded that both servant leadership and spiritual leadership have a positive effect on job satisfaction.

In this research, within the scope of “others” leadership styles, coach leadership, hr. specialist leadership, autocrat leadership, contingent, leadership, top management leadership, institutional leadership, empowering leadership, fair leadership, and democratic leadership styles were examined. It has been emphasized that these leadership styles are discussed in studies specific to higher education institutions, and that the relationship between academic staff’s job satisfaction and job satisfaction is positive in related studies (Haras, 2010; Muhonen, 2016; Alonderiene and Majauskaite, 2016; Lee et al., 2017; Rahman, 2018; Hee et al., 2020).

Based on this, the following hypothesis were developed in the research:

*H2*: Leadership style is a moderating variable for the positive relationship between leadership in HEI and academic staff’s job satisfaction.

*H2a*: There is a positive relationship between transformation leadership style in HEIs and academic staff’s job satisfaction.

*H2b*: There is a positive relationship between transactional leadership style in HEIs and academic staff’s job satisfaction.

*H2c*: There is a negative relationship between passive leadership style in HEIs and academic staff’s job satisfaction.

*H2d*: There is a positive relationship between servant leadership style in HEIs and academic staff’s job satisfaction.

*H2e*: There is a positive relationship between spiritual leadership style in HEIs and academic staff’s job satisfaction.

*H2f*: There is a positive relationship between other leadership styles in HEIs and academic staff’s job satisfaction.

Depending on many factors such as the level of economic development, management styles (Vliert and Einarsen, 2008), cultural values (Wu et al., 2018), individualistic-collectivist structure of employees (Hou, 2017), there are studies that show that leadership approaches differ on a country basis. Therefore, it is seen that different styles of leadership come to the fore in different geographical regions (Aycan et al., 2000; Vliert et al., 2010). For example, Mittal and Dorfman (2012) found that the dimensions of egalitarianism and empowerment are more important in European cultures than Asian cultures in their study examining the levels of servant leadership in different geographical regions. They stated that the dimensions of empathy and humility were more suitable for Asian cultures rather than European cultures. In addition, there are also studies that comparatively examine academic staff’s job satisfaction in different countries (Lacy and Sheehan, 1997; Bentley et al., 2013). For example, Lacy and Sheehan (1997) found in their study that academics in the United States (60%) were more satisfied with their jobs than academics in Hong Kong (50%). Bentley et al. (2013) determined the job satisfaction rate of academics in South Africa, located on the African continent, as 51%, the job satisfaction rate of academics in the USA as 61%, and the job satisfaction rate of academics in Finland, located in the European continent, as 67%. Based on all this literature, it is predicted that the continent of the country in which the academic staff work will be the moderator variable in their perceived leadership styles and job satisfaction and the following hypothesis were developed:

*H3*: The continent in which the countries are located is a moderating variable for the positive relationship between leadership in HEI and academic staff’s job satisfaction.

It has been stated in studies on a wide variety of organizational and national issues that there may be differences in different leadership preferences (Hofstede, 2001) and job satisfaction levels in societies that differ in terms of cultural values (Taras et al., 2010). In studies on leadership, it has been emphasized that collectivist and individualistic cultural values are important among social cultural values (Aycan et al., 2013). Triandis (1995) argues that leadership tends to be paternalistic and supportive in collectivist cultures, and achievement-oriented and participatory in individualistic cultures. According to House and Aditya (1997), “benevolent autocrat” leadership is the most admired leadership style in collectivist cultures. In a study, it was determined that employees with high collectivistic values perceived less mobbing when they perceived their managers as paternalistic leaders (Durmaz et al., 2020). Personal relationships are more important than duty in collectivist societies and personal relationships must be established first (Hofstede, 2001). Trust in institutions is established with the leader within personal relationships. An employee who trusts his leader is expected to have a positive job satisfaction (Shi et al., 2020; Zhou et al., 2022). Karadağ (2020), also mentions that because there is a stronger acceptance and respect for authority in collective cultures, leaders create more influence on these collective cultures than those in individual cultures. In line with all these research findings, it can be said that leadership is important in ensuring the job satisfaction of academic staff in collectivist cultures. The fact that institutions are seen as a family in collectivist cultures contributes to the employee’s developing a sense of loyalty to the institution and management (Saylık, 2017). As a result, it can be mentioned that the relationship between perceived leadership in higher education and job satisfaction in countries with collectivist cultures is higher than in countries with individualistic cultures (Aycan, 2006; Saylık, 2017; Durmaz et al., 2020). In line with the results of the relevant research, the following hypothesis has been developed:

*H4*: The positive relationship existing between leadership in HEIs, and academic staff’s is stronger in countries with collectivist cultures compared to countries with individualistcultures.

In a country, a high level of education affects development with a positive trend in terms of economic and social results, as it will create a qualified workforce. In this context, the evaluation of the education index in the HDI subcategory is important in terms of revealing the level of education, enabling comparison with different countries, and determining the measures and improving policies to be taken in countries with low education levels (Fırat et al., 2015). For example, in the context of job satisfaction, Blanchflower and Oswald (2005) stated that Australia, which ranked third in the HDI in 2004, ranks lower levels in the international job satisfaction rankings. In another study, cooperation in scientific publications, order of authorship, superiority and leadership in research activities were investigated between countries with different HDI. According to the results of this research, it is stated that the leadership characteristics of the authors participating from the countries with high HDI are more developed and they are especially responsible for the studies. It has been revealed that the authors of countries with medium and low levels of human development have a low level of leadership roles and show little participation as a corresponding author (González-Alcaide et al., 2017). In this context, it was predicted in the research that leadership styles in HEIs and job satisfaction in universities will also differ according to HDI variables.

*H5*: The positive relationship existing between leadership in HEI and academic staff’s is stronger in countries with very high/high human development indices (HDI) compared to countries with medium/low HDI.

## Materials and methods

### Research design

In this study, the meta-analysis method was used to determine the relation between leadership in HEIs and academic staff’s job satisfaction. Meta-analysis is a statistical method that aims to systematically bring together the quantitative findings of similar and independent studies on a specific subject in a consistent and coherent way according to selection criteria (Borenstein et al., 2009) and to reveal important moderator variables (Cohen et al., 2007; Dinçer, 2014).

### Study sample and selection criteria

Since publication bias is stated as an important negative factor in meta-analysis studies, it was preferred to use scientific articles and unpublished postgraduate theses in this study. The data used in the study are limited to January 2010–August 2022. The reason for the determination of this range can be shown as the increase of research on leadership in HEIs since 2010. It is also stated that the foundations of humanist leadership theories were laid (Karadağ, 2020). The reason why the research sample includes academic staff in higher education institutions can be cited as the frequent interactions between leaders and employees and the opportunity to examine the relationships between various variables as a result of these interactions (Syed et al., 2021). In addition, this sample was preferred to better understand the positive results of leadership styles to be exhibited in the academic environment (Li et al., 2022). The search process was carried out in English language by keywords and article texts or abstracts in all publications worldwide, between January 2010 and August 2022. Studies contain statistical information necessary for correlational meta-analysis (Pearson correlation values, sample size). Studies measure the relationships between leadership in HEIs and academic staff’s job satisfaction.

Clearly specifying the studies to be included in the meta-analysis in line with certain criteria and being consistent with the purpose of the research are important criteria to prevent publication bias (Berman and Parker, 2002). Therefore, first, a literature search was conducted in the Scopus, Web of Science, Proquest, and Ebsco databases to identify studies to be included in the meta-analysis. At this phase, the “leadership” term was taken as a base, and the terms “job satisfaction,” “faculty’s job satisfaction,” “faculty,” “academic staff’s job satisfaction,” “academic staff” OR “higher education” OR “university” OR “college” were used in the title, keywords, and abstract fields and searched in English. In line with this search model, 241 publications from ProQuest Dissertations and Theses database, 25 publications from Ebsco database, 152 publications from Web of Science database and 328 publications from Scopus database were reached. Thus, a total of 746 publications were reviewed for this study; A total of 215 publications describing the relationship between leadership and job satisfaction were included in the research. However, 44 of them were conducted in a qualitative study design. In 41 studies, Pearson correlation values were not specified; In 16 studies, the variable related to job satisfaction was not defined. In addition, it was determined that the sample of 54 studies consisted of both administrative and academic staff. Therefore, 155 studies were excluded from the analysis. In the second phase, the remaining 60 studies were analysed in detail 32 of these studies were excluded from the analysis because they were the same study which were in different databases; and 28 studies found appropriate to use in this study.

As a result of the examinations, a research sample including studies suitable for meta-analysis was obtained. Accordingly, there are 57 independent data sets obtained from 28 different studies in the study sample (Table 1).

**Table 1:** Frequency of the studies included in meta-analysis of the leadership in HEIs and academic staff’s job satisfaction.

Variables												Total
The year of studies		2010	2013	2014	2015	2016	2017	2018	2019	2020	
	*n*	2	1	2	4	5	2	3	2	6	28
Types of research		Article		Dissertations							
	*n*	15		13							28
The National Culture		Collectivist		Individualistic		UK					
	*n*	13		14		1					28
The Continent		Africa		America		Asia	Europe				
	*n*	3		11		11	3				28
The Human Development Index (HDI)		Low		Medium		High	Very high				
	*n*	2		2		5	19				28

When the descriptive statistics of the research included in the meta-analysis were examined, it was seen that 28 studies examining the relationships between leadership styles in HEI and academic staff’s job satisfaction were conducted in 9 studies between 2010 and 2015, 10 between 2016 and 2018, and 8 between 2019 and 2020. There is no study in 2021 and 2022. A total of 7,283 academic staff included in the sample.

Unpublished studies (i.e., dissertations) were also included in the study, since only the criticisms of including published articles in meta-analyses were considered. Of the 28 studies included in the research, 15 are articles and 13 are dissertations. Three studies in Africa (Nigeria, Uganda, South Africa), eleven studies in the Americas (United States), eleven studies in Asia (Pakistan = 2, Saudi Arabia = 2, Azerbaijan, Oman, Malaysia, Palestine, Iran, Indonesia), and three studies in Europe were conducted (Lithuania, Sweden and Turkiye).

### Coding procedure

Coding is a data extraction process in which clear data and data suitable for research are extracted from the information compiled in the studies (Karadağ, 2020). A coding form was created by the researchers to code the studies included in the meta-analysis process. In the coding form, (i) descriptive statistics and (ii) statistics of research variables were coded in Excel. Within the scope of descriptive statistics, the references of the research, the year it was published, the information about the sampling (sample size, the country in which the research was conducted, the cultural classification of the countries and the classification of the HDI of the countries), the names of the data collection tools were coded. Methodological analysis information and quantitative values (Pearson correlational values between leadership in HEI and academic staff’s job satisfaction) used within the scope of statistics of research variables are also defined. Coding was done in an appropriate way in the coding form. Thus, it is aimed to develop a special coding system specific to meta-analysis research that will examine the characteristics of both descriptive and research variables in detail.

### Moderator variables, analysis, and operational definitions

Moderator analysis is an analysis method used to test the direction of the differences between subgroups and the average effect sizes of the variables (Karadağ, 2020). The statistical significance of the difference between the moderator variables was tested using the *Q* statistical method developed by Hedges and Olkin (1985). In this method, the *Q*_b_ value was calculated to test the homogeneity between the groups (Kulinskaya et al., 2008; Borenstein et al., 2009). In the study, leadership styles, the continent, national culture and HDI variables were determined as moderator variables since they were thought to play a role in the average effect size.

The first moderator variable is leadership styles. In this research, moderators of leadership styles include: (i) transformational leadership, (ii) transactional leadership, (iii) passive leadership, (iv) servant leadership, (v) spiritual leadership and (vi) other. Other leadership styles discussed in the research are the studies gathered under the title of “other” and include the styles of leadership in which research based on a single frequency are found.

The second moderator variable, the continent where the research took place, was evaluated in terms of whether they were moderators in the relationship between leadership styles in HEI and academic staff’s job satisfaction. In this study, there are 6 studies from the African continent (3 countries), 22 studies from the Americas (11 countries), 20 studies from the Asian continent (11 countries), and 9 studies from the European continent (3 countries).

The third moderator variable is the national cultures of the countries (individualistic and collectivist cultures) named by Triandis and Gelfand (1998) and classified in Hofstede Insights (2020). People living in societies with an individualistic culture use their preferences within the social framework in the society; individuals in collectivist cultures meet the needs of their families and social frameworks before their own needs (Triandis, 1996) and shape their national cultures by preserving the integrity and order of the society Biddle (2012). In individualistic societies, individuals shape the society according to their own decisions and preferences and accept life as their own Biddle (2012). In collectivist societies, the services of individuals to society are taken as basis for social order and the life of individuals is seen as belonging to the society, they are a part of (Biddle, 2012). In line with all these views, the relationship between leadership in HEI and academic staff’s job satisfaction in countries with individualistic and collectivist cultures has been reviewed. In this study, of the 57 studies included in the national culture moderator analysis, 26 (*n* = 13) belong to a collectivist culture and 31 (*n* = 14) belong to an individualistic culture. The majority of research on individualistic culture has been carried out in the United States and European countries.

The fourth and final moderator variable is the current HDI, which expresses the economic, social, political and cultural processes (United Nations Development Programme (UNDP),2019) that expand individuals’ choices. In this meta-analysis study, the United Nations Development Programme (UNDP) (2019) is based on the HDI classification United Nations Development Programme (UNDP) (2019). Human development reports provide information and comments to eliminate general disadvantages in all countries in the world (Koçal, 2018). In the report, countries are classified as very high human development, high human development, medium human development, and low human development United Nations Development Programme (UNDP) (2019). Human development indices range from zero to one. The closeness of the index value to one is an indicator of very high human development. Considering the distribution of the research according to the HDI, it is seen that 42 studies have a very high index (19 countries), 6 studies a high index (5 countries), 4 studies a medium index (2 countries), and 5 studies a low index (2 countries).

### Effect size analyses

Effect size is a standard measure value used to determine the strength and direction of the relationship in the meta-analysis study (Borenstein et al., 2009). In this relational meta-analysis study, the effect size was calculated with the Pearson correlation coefficient (r).

There are two main models in the meta-analysis: the fixed effects model and the random effects model. In order to determine which model to use, it was taken into account whether the prerequisites of the model were met with the characteristics of the studies included in the meta-analysis (Kulinskaya et al., 2008; Borenstein et al., 2009). The fixed effects model includes the assumption that the study is functionally the same, and the goal is to estimate the effect size for a single defined population. If the study is believed to be unequal in functionality and generalizations are to be made over the estimated effect size for larger populations, the model to be used is the random effects model. In this study, a random effects model was applied in the meta-analysis processes when all conditions were taken into account. Comprehensive Meta-Analysis (CMA V 2) software was used in the meta-analysis processes.

### The common method bias

Various applications have been made in line with the recommendations in the literature to reduce the common method bias (Javed et al., 2020; Akhtar et al., 2021). First, Aslam et al. (2021) recommends stating the purpose of the research before applying the data collection tools and paying attention to the confidentiality and anonymity of the answers obtained from the data collection tools. When all the studies included in this meta-analysis study are examined, it can be said that confidentiality and anonymity are taken into account within the scope of the ethical dimension of the research and the purpose of the research is stated. Common method bias is also the case when a researcher creates estimates of validity and reliability that may lead a researcher to believe that a scale does not accurately reflect an implicit measure but does so accurately. Such an error may cause common method bias in future meta-analysis studies (Wall, 2014). As a result, the studies included in the meta-analysis were examined and it was seen that the data collection tools used were suitable for the purpose of the studies, and the validity and reliability information was presented. Thus, the existence of common method bias cannot be mentioned in this study.

### Statistical methods/analysis (reliability and validity of the study)

The reliability and validity of the results is considered one of the most important criteria in a meta-analysis. In this context, the steps for reliability and validity are as follows:

- In this study, while determining the inclusion and exclusion criteria, all the characteristics related to the field of study (leadership and job satisfaction) were evaluated together. The target set for job satisfaction is to evaluate the satisfaction of the academic staff with their jobs; It is not about assessing their life satisfaction.
- Since the studies included in the meta-analysis were not functionally equivalent, the random effects model was used.
- In this study, attention was paid to research sensitivity by including both published and unpublished studies to avoid publication bias. Also, no evidence of publication bias was observed with a funnel plot or tests. It was also determined that the effect size was not affected by publication bias.
- Coding reliability was performed to determine whether the studies in the coding form were coded correctly. For this purpose, two field experts experienced in meta-analysis studies were asked to recode approximately 17 studies, which were randomly selected and correspond to 30% of the studies included in the coding list, by adhering to the coding list created by the researchers. Cohen’s Kappa consistency coefficients, which were used in meta-analysis studies to determine the reliability of the coding form and to measure the reliability between raters (Leary, 2012), were calculated and the value was found to be 0.92 (*p* < 0.001). According to Landis and Koch (1977), this value indicates an “almost perfect” agreement between the coders.
- The basic condition for sampling in meta-analysis studies is that the sample best represents the population. The sampling is not expected to be the same as the population, as there are inclusion or exclusion criteria for sampling, and it consists of total errors that occur by chance. However, it is expected that an infinite number of studies will take place for meta-analysis in order for the sampling error to be zero (Karadağ, 2020). Therefore, considering that the sample of the studies included in the meta-analysis is not infinite; Random effects model was used in this study. In meta-analysis studies, correlation values are converted to “Fisher Z” values and analyses are performed on these values. While the analysis findings are being evaluated, they are interpreted by converting them into correlation coefficients. In correlation data, the correlation coefficient is used as the effect size in relation to the direction of the relationship (positive or negative). Correlation coefficient effect sizes are interpreted if it is between ±0.00 and ± 0.10, it is very weak; If it is between ±0.10 and 0.30, it is weak; between ±0.30 and 0.50 is moderate; ± 0.50 to 0.80 strong; ± 0.80 and above as a very strong effect (Cohen et al., 2007).

## Results

### Descriptive analysis

As can be seen in the forest plot examination (Supplementary Figure S1), all the random effect sizes for the correlation between leadership in HEIs and academic staff’s job satisfaction were significant (*p* < 0.05), and the confidence interval for each effect size did not cross zero.

Meta-analysis results between leadership in HEIs and academic staff’s job satisfaction are presented in Table 2. The findings support the H1 hypothesis, which states that there is a positive relationship between leadership in HEIs and academic staff’s job satisfaction. While the average effect size was determined to be *r* = 0.374, the lower bound value was calculated as *r* = 0.247 and the upper bound value as *r* = 0.504.

**Table 2:** Meta-analysis results related to relationship between leadership in HEIs and academic staff’s job satisfaction.

Variables	*K*	*N*	*r*	95% CI (Confidence Interval)		*Q*	*Q*_b_
				Lower Limit	Upper Limit		
Leadership and job satisfaction	57	7,283	0.374	0.247	0.502	2866.371*	
Moderator [Leadership style]							51.786*
Transformation	19		0.569*	0.392	0.746		
Transactional	13		0.265*	0.052	0.478		
Passive	9		−0.412*	−0.669	−0.156		
Servant	5		0.658*	0.313	1.003		
Spiritual	2		0.894*	0.354	1.435		
Other	9		0.632*	0.375	0.890		
Moderator [The continent]							6.219
America	22		0.273*	0.063	0.484		
Asia	20		0.316*	0.096	0.536		
Africa	6		0.373	−0.028	0.773		
Europe	9		0.754*	0.424	1.084		
Moderator [The national culture]							710
Collectivist	26		0.348*	0.157	0.538		
Individualistic	31		0.397*	0.221	0.573		
Moderator [Human development index]							0.682
Low	5		0.332	−0.110	0.774		
Medium	4		0.319	−0.175	0.812		
High	6		0.612*	0.207	1.016		
Very high	42		0.350*	0.197	0.504		

* *p* < 0.01.

In the other hypothesis sentences of the research; leadership styles, the continent in which the countries were located, the national culture and the HDI might be moderators for the relationship between leadership in HEIs and academic staff’s job satisfaction.

It is seen that the H2 hypothesis, which states that leadership styles have a moderator effect on the relationship between leadership in HEIs and academic staff’s job satisfaction, is supported (*Q*_b_ = 51.786 p < 0.05). From the leadership styles obtained from the studies included in the meta-analysis, spiritual leadership is very strong on the job satisfaction of the academic staff (*r* = 0.894); servant (*r* = 0.658), other (*r* = 0.632) and transformation (*r* = 0.569) leadership styles are strong on job satisfaction of academic staff; passive leadership (*r* = −0.412) has a medium effect on the job satisfaction of the academic staff, and transactional leadership (*r* = 0.265) has a weak effect on the job satisfaction of the academic staff.

H3, which asserted that the continent in which the countries are located was the moderating variable regarding the positive relationship between leadership in HEIs and academic staff’s job satisfaction, was not supported. In the moderator analysis performed, the positive relationship between leadership in HEIs and academic staff’s job satisfaction was not statistically significant (*Q*_b_ = 6.219, *p* > 0.05). Although the relationship difference was not statistically significant, teacher self-efficacy appears to have a positive relationship with academic staff’s job satisfaction in the continents of America (*r* = 0.273), Asia (*r* = 0.316), Africa (*r* = 0.373) and Europe (*r* = 0.754).

The findings did not support H4, which asserted that the national culture was a mediating variable for the positive relationship between leadership in HEIs and academic staff’s job satisfaction. In the moderator analysis performed, there was no significant difference between national culture [collectivist culture (*r* = 0.348) and individualistic culture (*r* = 0.397) (*Q*_b_ = 0.139; *p* > 0.05)].

H5, which expresses the role of The HDI as a moderator variable for the positive positive relationship between leadership in HEIs and academic staff’s job satisfaction was not supported. In the analysis of the moderator, the average effect size difference was found to be statistically insignificant (*Q*_b_ = 1.501, *p* > 0.05). Although the effect difference was not statistically significant, the relationship between leadership in HEIs and academic staff’s job satisfaction was in countries with low HDI (*r* = 0.332), medium HDI (*r* = 0.319), with high HDI (*r* = 0.612) and with very high HDI (*r* = 0.350).

### Publishing bias

**Figure 1:**
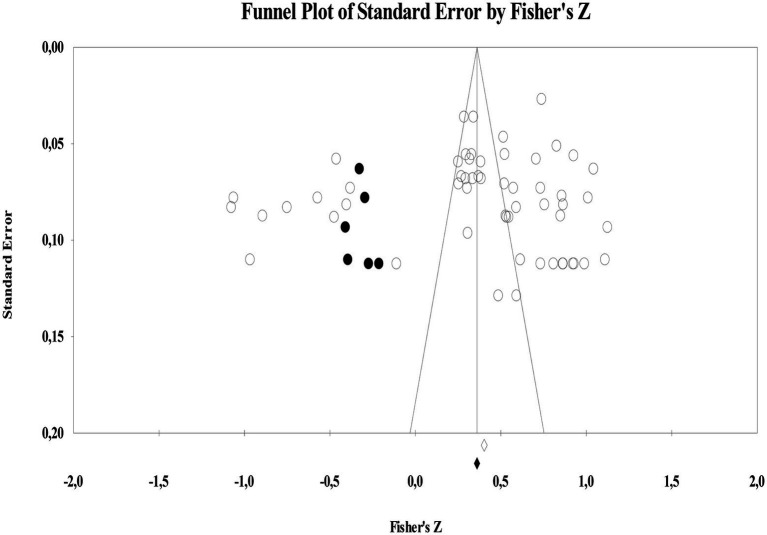
Funnel scatter plot illustrating relationship between leadership in HEIs and academic staff’s job satisfaction.

Since publications that produce meaningful results are included in the research process and negatively affect the analysis process, it is recommended to detect publication bias before starting the meta-analysis (Kalkan, 2017). The most commonly used method for publication bias is the funnel plot. The results of the funnel scatterplot showing the probability of publication bias of the studies included in the meta-analysis in this study are shown in Figure 1.

In case of any publication bias, the funnel plot is expected to be significantly asymmetrical. In particular, most of the studies included in the study are expected to be at the top of the figure and very close to the combined effect size. In line with all these indicators, it can be mentioned that no evidence of publication bias was observed in any of the 57 studies subjected to meta-analysis. However, since not all the individual effect sizes of the studies are symmetrical in the funnel, it is necessary to look at the publication bias statistics. Confidence tests showing the bias of the studies included in the meta-analysis and their results are given in Table 3.

**Table 3:** Confidence tests and results showing the bias of studies included in the meta-analysis.

Confidence tests	Data of confidence tests	
Rosenthal’s fail-safe N test	*Z*-value for the studies reviewed	39.493
	*p*-value for studies reviewed	0.000
	Alpha	0.050
	Direction	2
	*Z*-value for Alpha	1.959
	Number of observed studies	57
	Fail- Safe N (FSN)	3,087
Begg ve Mazumdar Rank Correlation	Tau	0.043
	*Z*-value for tau	0.474
	*p*-value (with 1 tail)	0.317
	*p*-value (2-tailed)	0.634
Egger’s regression Test	Standard Error	2.703
	%95 lower Limit (1 tailed)	−8.100
	%95 Upper Limit (2 tailed)	2.737
	*t*-value	0.991
	sd	55
	*p*-value (with 1 tail)	0.162
	*p*-value (2-tailed)	0.325

As seen in Table 3, the results of Rosenthal’s Fail-Safe N Test reveal that the meta-analysis result is statistically significant (*p* = 0.000). In other words, to eliminate the significance of meta-analysis results, 3,087 studies with zero effect size value are needed (*Z* value = 39.493; *p* < 0.00; alfa value = 0.05). The fact that Kendall’s Tau coefficient obtained from Begg and Mazumdar Rank Correlations is not statistically significant (Tau = 0.043; *z* value for Tau = 0.474; *p* value (1-tailed) = 0.317; p value (2-tailed) = 0.634) is an indication that there is no publication bias. From the result of Egger’s Linear Regression method (*p* = 0.325 > 0.05), it can be stated with 95% confidence that there is no publication bias. According to the results of Rosenthal’s Fail-Safe N Test, Begg and Mazumdar Rank Correlations, and Egger’s Linear Regression method, which were used to determine the validity and publication bias of the research, it was concluded that the publication bias was low. In this study, publication bias was tested also using Duval and Tweedie’s trim and fill tests in Table 4.

**Table 4:** The results of Duval and Tweedie’s trim and fill tests.

	Excluding study	Point estimate	CI (confidence interval)		*Q*
			Lower limit	Upper limit	
Observed values		0.37	0.24	0.50	2866.37
Adjustment values	0	0.37	0.24	0.50	2866.37

When Duval and Tweedie’s trim and fill tests results in Table 4 are examined, it is seen that there is no significant difference between the observed effect and the artificial effect determined to adjust for the effect that may arise from publication bias. The difference between the fixed effect size and the observed effect size is zero, since there is no missing data on both sides of the centerline and the studies concentrated on both sides show a symmetrical distribution (Coğaltay et al., 2014).

## Discussion and conclusion

In the current study, that was conducted to examine the relation leadership in HEIs and academic staff’s job satisfaction, using the meta-analysis method, the overall effect size of 57 different studies that were determined in accordance with the selection criteria was calculated. In addition, it was aiming to answer the question if there was a significant relation between the parameters according to the moderator variables (leadership theories, the continent where the research was conducted, national culture and HDI).

The first finding of the study is that there is a moderate and direct relation between leadership in HEIs and academic staff’s job satisfaction. As the related studies in the literature are examined, it is stated that leadership in HEIs is related to academic staff’s job satisfaction (Alonderiene and Majauskaite, 2016; Harris et al., 2016; Barnett, 2018; Liu et al., 2021). Academic staff should be satisfied with their jobs to fulfil their educational, research-investigation-based, and social responsibilities such as teaching, designing practice hours for the course material, conducting scientific studies, and carrying out projects. There is a direct relation between the academic staff’s job satisfaction and the program/education outcomes, the higher the satisfaction level of the academic staff’ results with the greater the program/education outcomes. High leadership behaviours exhibited by academic staff’ are also considered important on academic staff’s job satisfaction. Academic staff’ due to their position as scientists are expected to be able to lead, influence, stimulate the society while being open to communication and permissive (Caglar, 2004). This identity can be accepted as an indicator of the job satisfaction of the academic staff in terms of developing the vision of the university and producing a sense of belonging, as well as their leadership styes.

In this research, it has been determined that leadership styles are moderators in the relation between leadership in HEIs and academic staff’s job satisfaction. According to this finding, the effect of spiritual leadership on the relationship between the academic staff’s job satisfaction and the leadership in HEI is at the highest level; It has been determined that servant, other and transformational leadership styles have positive and strong effects. Moreover, passive leadership has negative and moderate effects while transactional leadership has positive but weak effects on the relation between leadership in HEIs and academic staff’s job satisfaction.

It is an expected result that the effect of leadership styles on the relation between leadership in HEIs and academic staff’s job satisfaction is direct and significant. In this study, it was determined that there is a positive and high level of relationship between spiritual leadership and academic staff job satisfaction. Spiritual leadership emphasizes the spiritual side of people, and it is seen that spiritual leaders emphasize issues such as love, compassion, honesty, harmony, unity, and peace (Polat, 2011). Moreover, it is stated that managers who show spiritual leadership characteristics are adored by their employees which is in direct relation with job satisfaction expectations (Pio and Tampi, 2018; Maryati et al., 2019; Djaelani et al., 2021). The fact that leaders in HEIs also have strong spiritual feelings towards the institution can positively affect their job satisfaction.

In this study, it was determined that there is a positive and high level of relationship between servant leadership and academic staff job satisfaction. It is also stated in the literature that servant leaders, who have the characteristics of helping the success and development of the employees in the institution and dedicating themselves to developing the vision of the institution, increase the job satisfaction of the individuals working in the institution (Amah, 2018; Zargar et al., 2019; Adiguzel et al., 2020). It is expected that the presence of a manager who supports their employees within the organization will have an impact on the job satisfaction of the employees’. Likewise, a leader in HEIs who is devoted to the institution and who aims to develop the vision of the institution and whose servant-leader characteristics dominate is expected to have a high levels of job satisfaction.

In the study, it was determined that that there is a positive and high level of relationship between transformational leadership and academic staff job satisfaction. Many studies examining leadership styles and job satisfaction in higher education have concluded that there is a moderate and positive relationship between transformational leadership and academic staff job satisfaction (Duyan, 2019; Gölebakar, 2020). Transformational leaders aim to change the perceptions of the employees in the organization by way of variety of activities by putting their employees in the center of the activities stemming a high levels of job satisfaction within the institution (Cote, 2017). It can be said that leaders in HEIs displaying transformational leadership styles and taking their own interests and needs as the basis while achieving their goals they focus on will increase their job satisfaction.

In this research, it has been determined that passive leadership has negative and moderate levels effect on academic staff job satisfaction and transactional leadership has a positive and low-level effect on academic staff job satisfaction. Passive leadership is a leadership style in which the leader does not interfere with the process and avoids talking to employees or setting the desired standards (Bass et al., 2003). Transactional leadership, on the other hand, is defined as a process based on mutual interests between the leader and the employee, in which employees gain prestige and wages as a result of meeting the expectations of the leaders (Isa et al., 2011). It is inevitable that both leadership characteristics will have lower effects on job satisfaction than other leadership styles. As a matter of fact, it is stated in the literature that the relation between passive and transactional leadership and job satisfaction is low, and there is even a negative relation (Nguni et al., 2006; Nazim and Mahmood, 2018). In this manner, it can be said that leaders’ acting with a certain salary or extrinsic motivation or hiding their leadership characteristics have an insignificant effect on their job satisfaction or that the effect is less than those with other leadership styles.

No statistically significant difference was observed in the relation between leadership in HEIs and academic staff’s job satisfaction in any of the four continents within the scope of the research. Owing to globalization in the 21st century, it is an expected result that leadership and job satisfaction are expected to be high among the characteristics of the teaching staff independent of geographical locations. Although the continent variable was determined as the moderator variable for the relationship between leadership n HEIs and job satisfaction, it was determined that continent was not a significant variable in this study. It is possible to state that there are studies with similar findings in the literature however, there are more studies that conclude that continent is a significant variable (Hou, 2017; Wu et.al., 2018; Neubert et al., 2022). There might be different reasons for this. First of all, it was aimed to reveal cultural, economic and social differences while determining the continent variable as a moderator variable. Since the sample size that could detect national differences in the research universe could not be reached, it is thought that these dimensions should be compared with a larger sample set in future studies, although universities operate in different geographies, it is thought that this has led to such a result because they are in a similar organizational structure. Since the structure of universities does not change radically on a geographical basis, it is thought that continental difference does not have a significant moderator effect on the relationship between leadership n HEIs and job satisfaction.

In this study, it was determined that the relationship between leadership in HEIs and academic staff’s job satisfaction did not differ according to countries with collectivist and individualistic society. Although a society’s being in an individualistic or collectivist culture gives information about the individuals, institutions, behaviours and functioning of those institutions (Carıkcı and Koyuncu, 2010); Individualist and collectivist cultures cannot always exhibit a homogeneous structure, both at the social and institutional level. Even within the same country or society, a heterogeneous structure is exhibited in terms of cultural approach (Hofstede et al., 2010; Keçeci, 2017). There are different findings about individualism–collectivism and job satisfaction in the literature. Hui et al. (1995) found that job satisfaction is higher in collectivist societies. Nevertheless, Harrison (1995), Griffeth and Hom (1987), and Lincoln and Kalleberg (1985) reported that employees in individualistic cultures have higher job satisfaction. Although there are studies stating that leadership styles (spiritual, paternalistic, educational) are higher in collectivist cultures than in individualistic cultures (Novikov, 2017; Saylik, 2017; Karadağ, 2020). In his research, Saylik (2017) concluded that there is no significant relationship between collectivism and leadership styles emphasizing authoritarianism, interventionism, and insufficiency. Similar research findings, which determined that the relationship between leadership styles in academic organizations and academic staff job satisfaction, do not differ according to countries with collectivist and individualistic society structures, also support the findings of this research (Durmaz et al., 2020). As a result, it can be said that both cultural structures can affect the leadership in HEIs and academic staff’s job satisfaction direct or reverse from different aspects.

Likewise, it was determined that HDI types were not moderators in the relation between leadership in HEIs and academic staff’s job satisfaction. Among the countries included in the research, it can be said that the academic staff working in different countries in terms of HDI find the profession of academics valuable, they are satisfied with their work and their perceptions of leadership in HEIs are high. Although Blanchflower and Oswald (2005) found in their research in Austria that their country has a high HDI index, the job satisfaction of the employees found at low level. However, Hamutoğlu et al. (2020), found that all employees in higher education institutions in Norway with a high HDI index are satisfied with their institutions. Although there are differences in the literature, it can be said that academic staff working in countries with different levels of human development find their profession valuable and are satisfied with their job. As a result, it can be accepted that the relationship between academic staff’s perceived leadership styles and job satisfaction does not differ significantly according to the level of human development.

### Limitations and suggestions for future research

The current study was conducted using data obtained from primary sources. The major disadvantage of the current research is the possibly relational nature of the studies from which the data were obtained. Considering that qualitative studies are more effective in explaining the nature of leadership in HEIs, claiming that the obtained results can fully explain the causal effects would be biased. In addition, the fact that most of the studies on the academic staff’s job satisfaction levels of leadership in HEIs are correlational indicates the existence of a potential method bias.

Despite the strategies developed to access the studies to be included in the current meta-analysis, it was not possible to reach all studies. It can be explained with the fact that the full texts of some studies could not be accessed through the searched databases can be cited. Hence, some studies that are thought to contain data suitable for the current research could not be reached. Although there were no statistical results indicating publication bias, the absence of publication bias could not be guaranteed as unpublished studies were not accessible. Secondly, in this study studies reporting the correlation coefficient (r) were included in the meta-analysis. Therefore, researchers may be advised to report the findings that led to the meta-analysis, rather than providing a single conclusion. Thirdly, since the publication language of the studies included in the current research was limited to English, studies published in other languages could not be reached. Thus, most of the included studies were conducted in various states of the United States. Further meta-analysis studies should consider studies published in different languages to reveal cultural differences. Another limitation of the study is that the sample of the present study consists of studies published between 2010 and 2022. Accordingly, this limitation should be considered when generalizing the results obtained.

Due to the positive relations between the leadership in HEIs and academic staff’s job satisfaction, it may be recommended to give trainings to the faculty to improve their leadership skills within the institution. In addition, it can be suggested that scientific studies that reveal the effects that increase the job satisfaction of the academic staff should be periodically updated and measures should be taken to increase the job satisfaction within the institution. It is recommended that all findings required for inclusion of individual studies in such meta-analysis studies should be reported by the researchers. For future studies, it is recommended to conduct studies examining similar variables based on the findings of international reports that allow OECD countries to be compared in terms of education.

### Theoretical implications

Theoretically, this research confirmed that the relationship between leadership in higher education and job satisfaction is positive. It has contributed to the importance of leadership styles in higher education in ensuring the job satisfaction of academic staff. It has been revealed that when academic staff are recognized, supported and rewarded by university administrators, their job satisfaction levels will tend to increase. Therefore, the leadership style of university administrators will contribute to the job satisfaction of academic staff. This research has mentioned on the importance of leadership styles adopted in higher education institutions in theory in recent years. Thus, future research will contribute to the further growth and integrative potentials of these leadership types.

### Practical implications

This research provides policy makers, practitioners, and administrators with relevant information in a variety of ways. According to the findings of the research, firstly, spiritual leadership should be adopted by the academic staff in order to ensure job satisfaction. It is necessary to adopt a leadership approach that will consider the emotional, spiritual and mental needs of academic staff in higher education institutions. Thus, the learning, research and teaching climate in higher education institutions can be positively affected. Administrators in higher education should develop an academic organizational structure inspired by a new and strong culture that will meet all the needs, desires and aspirations of academic staff. Servant leadership is another leadership that academic staff should adopt to ensure job satisfaction. It may be beneficial to develop leadership training programs that listen to and care for academics’ needs and try to assist their career development. In this case, higher education institutions should try to create an open, sincere, and honest workplace in order to ensure the job satisfaction of their academic staff. A friendly academic environment enables teaching staff to make the profession an enjoyable career. Moreover, it can be suggested to raise awareness of administrators and academic staff working in higher education institutions by giving trainings on the importance of servant leadership. The findings showed that it is beneficial for academic staff to develop transformational leadership skills to increase job satisfaction. For the academic staff to be more productive and achieve high performance, the presence of more transformational leaders in the institution can be recommended. In an academic environment where the competencies of academic staff are evaluated and rewarded, academic staff who research and teach, might be highly motivated and less likely to seek new jobs. In summary, university administrators who adopt transformational leadership should create an academic environment where innovative and creative thinking abilities are encouraged and valued.

The changing leadership roles of administrators, who will increase the job satisfaction of academic staff in higher education institutions in the future, will be an indispensable and important subject of future research. This research shows that humanist leadership roles rather than traditional leadership roles are important in increasing academician job satisfaction in today’s higher education institutions. In-depth research is needed to understand the basis of these positive reactions to spiritual, servant and transformational leadership roles in higher education institutions.

## Data availability statement

The raw data supporting the conclusions of this article will be made available by the authors, without undue reservation.

## Author contributions

GK and BG designed the study, reviewed the literature, organized the database, and performed the meta analysis of manuscript. BG, VE, and ÇA contributed to introduction. GK wrote findings, results, and conclusion of the manuscript. GT reviewed literature and edited the manuscript and references. All authors have read and approved the submitted version.

## Conflict of interest

The authors declare that the research was conducted in the absence of any commercial or financial relationships that could be construed as a potential conflict of interest.

## Publisher’s note

All claims expressed in this article are solely those of the authors and do not necessarily represent those of their affiliated organizations, or those of the publisher, the editors and the reviewers. Any product that may be evaluated in this article, or claim that may be made by its manufacturer, is not guaranteed or endorsed by the publisher.

## Supplementary material

The Supplementary material for this article can be found online at: https://www.frontiersin.org/articles/10.3389/fpsyg.2022.1038824/full#supplementary-material
