# Supplementary material for: The relation between leadership styles in higher education institutions and academic staff’s job satisfaction: A meta-analysis study
Source: Front Psychol. 2022 Nov 17;13:1038824. doi: 10.3389/fpsyg.2022.1038824 (PMC9714620; doi:10.3389/fpsyg.2022.1038824)
Supplement: Supplementary file 1 [file Data_Sheet_1.docx]

Appendix 1. References of research included in the meta-analysis

Aboramadan, M., Dahleez, K., and Hamad, M. H. (2020). Servant leadership and academics outcomes in higher education: the role of job satisfaction. *International Journal of Organizational Analysis.* 29:3, 562-584. doi: 10.1108/IJOA-11-2019-1923

Abbas, G., Khalily, M. T., and Riaz, M. N. (2016). Mediating role of work-related attitudes between leadership styles and well-being. *Pakistan Journal of Commerce and Social Sciences (PJCSS),*10:2, 257-273.

Alonderiene, R., and Majauskaite, M. (2016). Leadership style and job satisfaction in higher education institutions. *International Journal of Educational Management,* 30:1, 140-164. doi: 10.1108/IJEM-08-2014-0106

Ali, N., Jan, S., Ali, A., and Tariq, M. (2014). Transformational and transactional leadership as predictors of job satisfaction, commitment, perceived performance and turnover intention (empirical evidence from Malakand division, Pakistan). *Life Science Journal,* 11:5, 48-53.

Al-Maqbali, F. H. A. (2017). *The impact of the leadership styles of Deans on the Academic staff’ level of job satisfaction in nursing education in Oman [*dissertation*],* University of Glasgow.

Alquhaiz, K. (2020). *Academic leadership styles and academic staff’ job satisfaction at the King Saud University* [dissertation] Concordia University Chicago.

Alsunaydi, R. A. (2020). *The relationship between department chairs’ leadership style and academic staff’ job satisfaction at King Saud University in Saudi Arabia*[dissertation] University of the Incarnate Word.

Asgari, A., Mezginejad, S., and Taherpour, F. (2020). The role of leadership styles in organizational citizenship behavior through the mediation of perceived organizational support and job satisfaction. *Innovar,*30:75, 87-98. doi: 10.15446/innovar.v30n75.83259

Atwood, D.E. (2018). *Quantitative analysis of full-range leadership on subordinate employee job satisfaction and supervisor effectiveness in higher education.* [dissertation] Northcentral University

Blasetti, S. J. (2020). *The relationship between transformational leadership, job satisfaction and work-life balance among California Community College adjunct (Part-Time) Faculty Counselors* [dissertation] The Chicago School of Professional Psychology.

DeWoody, S. D. (2016). *The relationship between servant leadership and faculty satisfaction among academic staff in the council of independent colleges.* [dissertation] Dallas Baptist University.

Farris, J. D. (2010). *Servant leadership in Alabama’s regional public universities: The president’s role in fostering job satisfaction*. [dissertation] Alabama State University.

Harash, A. H. (2010). *An analysis of the relationship between the perceived leadership styles of educational leaders and the job satisfaction of academic staff who serve under them within community colleges*. [dissertation] Pepperdine University.

Harris, K., Hinds, L., Manansingh, S., Rubino, M., and Morote, E. S. (2016). What type of leadership in higher education promotes job satisfaction and increases retention? *Journal for Leadership and Instruction,*15:1, 27-32.

Hee, O. C., Shi, C. H., Kowang, T. O., Fei, G. C., and Ping, L. L. (2020). Factors influencing job satisfaction among academic staffs. *International Journal of Evaluation and Research in Education,*9:2, 285-291. doi: 10.11591/ijere.v9i2.20509

Jastrow, E. L. (2019). *Impact of perceived transformational leadership on turnover intention in prelicensure nursing faculty* [dissertation] Grand Canyon University.

Mwesigwa, R., Tusiime, I., and Ssekiziyivu, B. (2020). Leadership styles, job satisfaction and organizational commitment among academic staff in public universities. *Journal of Management Development,* 39:2. 253-268. doi: 10.1108/JMD-02-2018-0055

Muhonen, T. (2016). Exploring gender harassment among university teachers and researchers. *Journal of Applied Research in Higher Education,*8:1, 131-142. doi: 10.1108/JARHE-04-2015-0026

Perey, J. D. (2015). *Transformational leadership and employee satisfaction in two-year postsecondary institutions in rural Arizona*. [dissertation] Grand Canyon University.

Ragaisis, J. A. (2018). *The influence of servant leadership and transformational leadership on faculty job satisfaction and performance in higher education* [dissertation] Concordia University Irvine.

Rahman, M. A. (2018). Influence of female leadership styles and organization culture on locus of control and job satisfaction. *Integrated Journal of Business and Economics,* 2:2,123-134. doi: 10.33019/ijbe.v2i2.76

Robyn, A., and Du Preez, R. (2013). Intention to quit amongst generation Y academics in higher education. *SA Journal of industrial Psychology,*39:1, 1-14. doi: 10.4102/sajip.v39i1.1106

Sakiru, O. K., Othman, J., Silong, A. D., Kareem, S. D., Oluwafemi, A. O., and Yusuf, G. O. (2014). Relationship between head of department leadership styles and lecturers job satisfactions in Nigerian public universities. *Asian Social Science,*10:6, 138-144. doi: 10.5539/ass.v10n6p138

Townsend, A. (2016). *The impact of perceived transformational leadership style on nursing faculty satisfaction* [dissertation] Northcentral University.

Yıldız, I. G., and Şimşek, Ö. F. (2016). Different pathways from transformational leadership to job satisfaction: The competing mediator roles of trust and self‐efficacy. *Nonprofit Management and Leadership,*27:1, 59-77. doi: 10.1002/nml.21229

Worthy, k. (2015). *Relationships among leadership styles and job satisfaction levels: a national study of college of nursing faculty and deans in research intensive institutions.*[dissertation] University of South Carolina
