# Supplementary figures and images for: The relation between leadership styles in higher education institutions and academic staff’s job satisfaction: A meta-analysis study
Source: Front Psychol. 2022 Nov 17;13:1038824. doi: 10.3389/fpsyg.2022.1038824 (PMC9714620; doi:10.3389/fpsyg.2022.1038824)

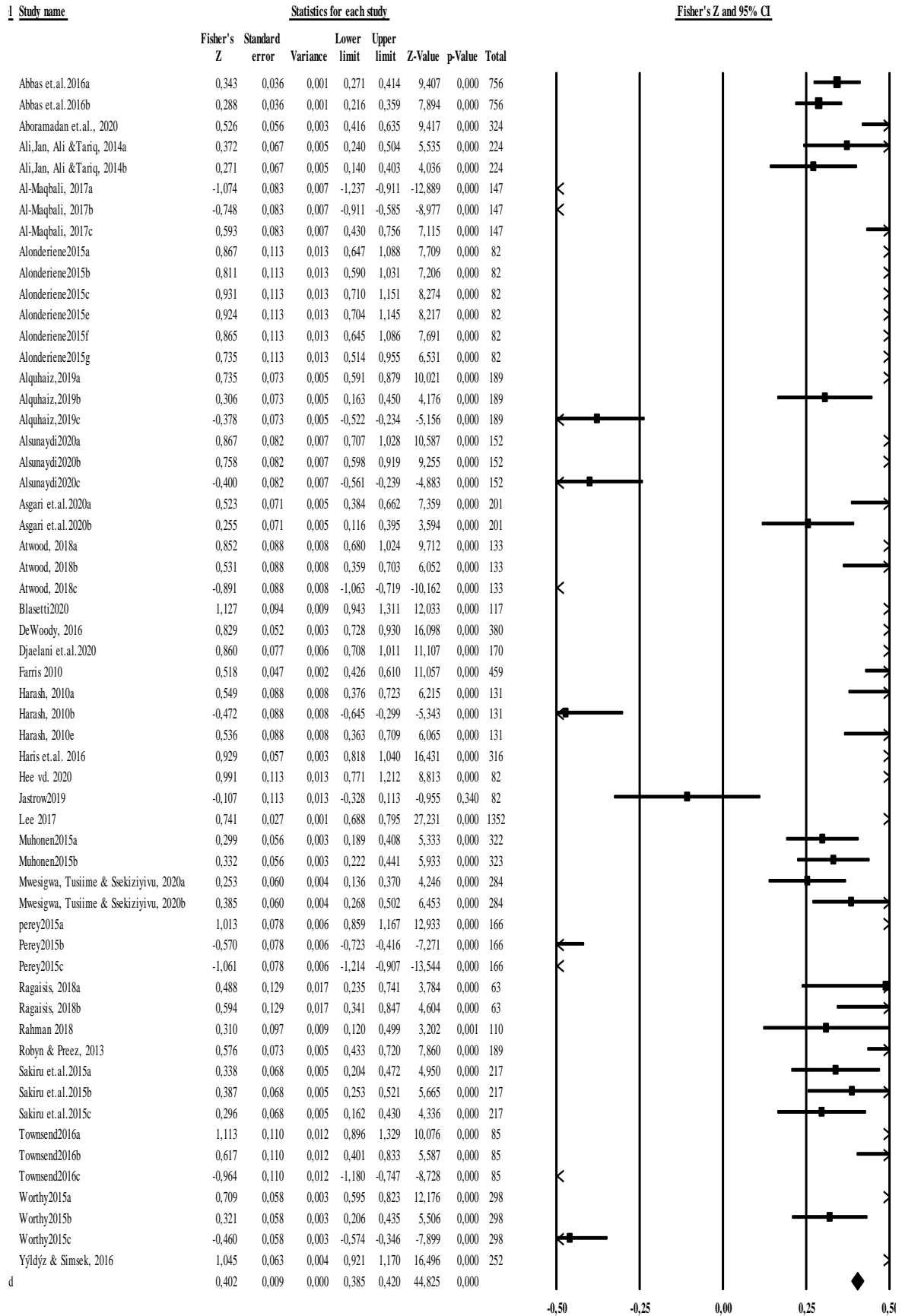

Figure 1. Forest plot of research included in meta-analysis

Supplement: Supplementary file 2 [file Image_1.pdf]
